# A Retrospective Overview of Zika Virus Evolution in the Midwest of Brazil

**DOI:** 10.1128/spectrum.00155-22

**Published:** 2022-03-07

**Authors:** Marta Giovanetti, Luiz Augusto Pereira, Talita Émile Ribeiro Adelino, Vagner Fonseca, Joilson Xavier, Allison de Araújo Fabri, Svetoslav Nanev Slavov, Poliana da Silva Lemos, William de Almeida Marques, Simone Kashima, José Lourenço, Tulio de Oliveira, Carlos Frederico Campelo de Albuquerque, Carla Freitas, Cassio Roberto Leonel Peterka, Rivaldo Venancio da Cunha, Ana Flávia Mendonça, Vinícius Lemes da Silva, Luiz Carlos Junior Alcantara

**Affiliations:** a Laboratório de Flavivírus, Instituto Oswaldo Cruz, Fundação Oswaldo Cruz, Rio de Janeiro, Rio de Janeiro, Brazil; b Laboratório de Genética Celular e Molecular, Instituto de Ciências Biológicas, Universidade Federal de Minas Gerais, Belo Horizonte, Minas Gerais, Brazil; c Laboratório Central de Saúde Pública Dr. Giovanni Cysneiros, Goiânia, Goiás, Brazil; d Laboratório Central de Saúde Pública do Estado de Minas Gerais, Fundação Ezequiel Dias, Belo Horizonte, Minas Gerais, Brazil; e Organização Pan-Americana da Saúde/Organização Mundial da Saúde, Brasília, Brazil; f University of São Paulo, Ribeirão Preto Medical School, Blood Center of Ribeirão Preto, Ribeirão Preto, São Paulo, Brazil; g Coordenação Geral das Arboviroses, Secretaria de Vigilância em Saúde/Ministério da Saúde (CGARB/SVS-MS), Brasília, Brazil; h Department of Zoology, Peter Medawar Building, University of Oxford, Oxford, UK; i School for Data Science and Computational Thinking, Faculty of Science and Faculty of Medicine and Health Sciences, Stellenbosch University, Stellenbosch, South Africa; j Coordenação Geral de Laboratórios de Saúde Pública/Secretaria de Vigilância em Saúde, Ministério da Saúde (CGLAB/SVS-MS), Brasília, Brazil; k Fundação Oswaldo Cruz, Bio-Manguinhos, Rio de Janeiro, Rio de Janeiro, Brazil; Forschungszentrum Jülich GmbH

**Keywords:** Zika virus, Asian lineage, Midwest Brazil, genomic epidemiology

## Abstract

Since the introduction of the Zika virus (ZIKV) into Brazil in 2015, its transmission dynamics have been intensively studied in many parts of the country, although much is still unknown about its circulation in the midwestern states. Here, using nanopore technology, we obtained 23 novel partial and near-complete ZIKV genomes from the state of Goiás, located in the Midwest of Brazil. Genomic, phylogenetic, and epidemiological approaches were used to retrospectively explore the spatiotemporal evolution of the ZIKV-Asian genotype in this region. As a likely consequence of a gradual accumulation of herd immunity, epidemiological data revealed a decline in the number of reported cases over 2018 to 2021. Phylogenetic reconstructions revealed that multiple independent introductions of the Asian lineage have occurred in Goiás over time and revealed a complex transmission dynamic between epidemic seasons. Together, our results highlight the utility of genomic, epidemiological, and evolutionary methods to understand mosquito-borne epidemics.

**IMPORTANCE** Despite the considerable morbidity and mortality of arboviral infections in Brazil, such as Zika, chikungunya, dengue fever, and yellow fever, our understanding of these outbreaks is hampered by the limited availability of genomic data to track and control the epidemic. In this study, we provide a retrospective reconstruction of the Zika virus transmission dynamics in the state of Goiás by analyzing genomic data from areas in Midwest Brazil not covered by other previous studies. Our study provides an understanding of how ZIKV initiates transmission in this region and reveals a complex transmission dynamic between epidemic seasons. Together, our results highlight the utility of genomic, epidemiological, and evolutionary methods to understand mosquito-borne epidemics, revealing how this toolkit can be used to help policymakers prioritize areas to be targeted, especially in the context of finite public health resources.

## INTRODUCTION

The Zika virus (ZIKV) is a mosquito-borne flavivirus that was first identified in Uganda in 1947 (1). Outbreaks of ZIKV infection have already been recorded in Africa, Asia, the Pacific, and the Americas (2, 3). The first confirmed case of ZIKV infection in the Americas was reported in Northeast Brazil in May 2015 (4), although phylogenetic studies indicate virus introduction much earlier (2013 to 2014) (5). Since then, the virus has spread throughout the Americas, probably due to a combination of several factors, including a completely susceptible population, favorable climatic conditions for the adequability of the Aedes aegypti mosquitoes as main vectors for its transmission, and sustained human mobility (6–8). Between January 2016 and December 2018, the Brazilian Midwestern region, which covers an area of 1.6 million km^2^ and is inhabited by about 14 million people in 467 municipalities, reported a total number of 54,457 Zika cases (9–14). Most of these cases (55%) were reported in the states of Mato Grosso and Goiás, across several epidemic seasons (9–14). Despite some work done over the large epidemic between 2015 and 2016, there is still a paucity of studies directly investigating the circulation and genetic diversity of the ZIKV in this region. In this study, using our experience with mobile laboratory (15), we used nanopore sequencing to generate ZIKV genomes from infected patients residing in Goiás and provide a retrospective reconstruction of its transmission dynamics in that state.

## RESULTS

**FIG 1:**
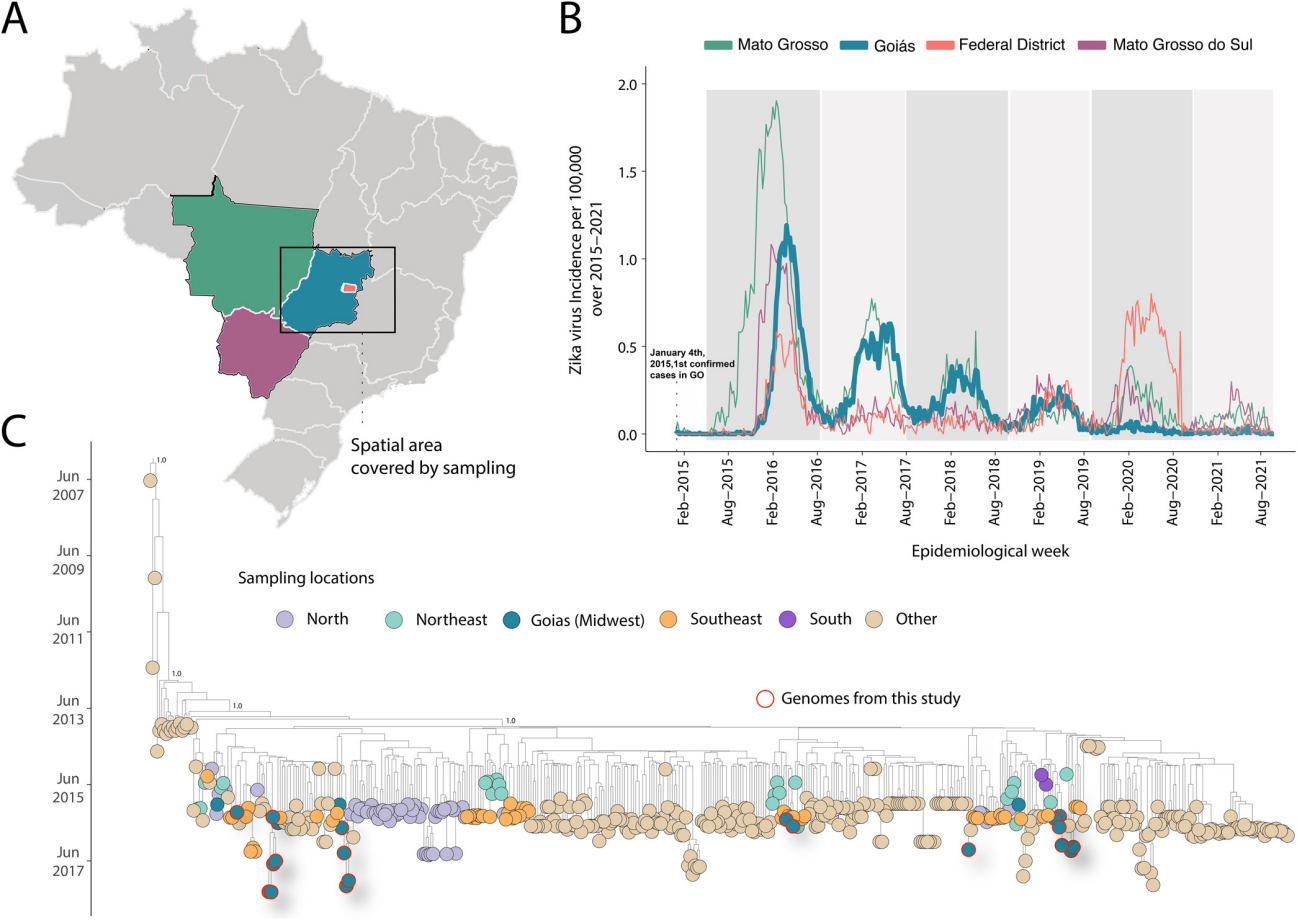
Genomic epidemiology of ZIKV in Midwest Brazil. (A) Map of Brazil showing the spatial area under investigation. (B) Weekly notified Zika cases normalized per 100,000 individuals in in the Brazilian Midwest region (Federal District and the states of Mato Grosso, Goiás, and Mato Grosso do Sul) between 2015 and 2021. Epidemic curves are colored according to geographical locations. Incidence (cases per 100,000 population) is presented in log_10_ for visual purposes. (C) Time-scaled maximum clade credibility tree of ZIKV-Asian lineage in Brazil, including the 23 new genomes generated in this study plus *n* = 479 reference strains sampled worldwide. Tips are colored according to the sample source location. Values around nodes represent posterior probability support of the tree nodes inferred under Bayesian evolutionary analysis using a molecular clock approach.

The 23 sequenced samples obtained in this study were collected from females (65%) and males (35%) (Table S1) with a median age of 30 years (range: 19 to 57). All sequenced samples were collected from different municipalities in the state of Goiás (Table S1, Fig. 1A) and contained sufficient viral genetic material (≥2 ng/μL) for library preparation. Cycle threshold (*C_T_*) values were on average 27.96 (range: 25 to 32), and sequences presented a median genome coverage of 82.5% (range: 56.1 to 93.2). Epidemiological data and sequencing statistics are detailed in Table S1.

Figure 1B shows the ZIKV weekly cases normalized per 100,000 individuals notified between 2015 and 2021 in the Brazilian Midwest region (Federal District and the states of Mato Grosso, Goiás, and Mato Grosso do Sul). This weekly reported incidence revealed a major outbreak in the Midwest region during 2015 to 2016, after which ever smaller epidemics took place over the years but the virus persisted through year-round transmission cycles. Overall, the state of Goiás reported the lowest incidence in recent years (2020 to 2021). Interestingly, the Federal District, which experienced the smallest initial outbreaks in 2015 to 2016, later presented a temporary resurgence in 2019 to 2020 (Fig. 1B). Over this period, the cumulative number of cases per 100,000 was 17 for the state of Goiás, 36 for the state of Mato Grosso, and 35 for the state of Mato Grosso do Sul. Although we did not assess the factors dictating the general trend in decreasing incidence over the years, it is likely to have been mediated by the accumulating herd immunity in the region since the virus’s introduction (16). Indeed, some studies have demonstrated this effect in other Brazilian states (16, 17).

To accurately establish evolutionary relationships among the newly generated sequences and other known ZIKV isolates, we subjected a combined data set to phylogenetic inference. A regression of genetic divergence from root to tip against sampling dates confirmed sufficient temporal signal (coefficient correlation = 0.70, *r*^2^ = 0.50). Our maximum clade credibility (MCC) tree showed that the newly sequences obtained in this study are scattered throughout the tree and clustered together with viral strains isolated in other Brazilian regions (northeastern and southeastern), suggesting that those regions have likely acted as steppingstone spots for the dissemination of the virus into the state of Goiás (Fig. 1C), which might have been influenced by the increased human mobility and vector suitability. From our time-measured tree, we estimated the time of the most recent common ancestor (TMRCA) to have occurred between mid-February 2014 (95% highest posterior density ranging from 10 February 2014 to 10 October 2014) for the first introduction event and late November 2016 (95% highest posterior density ranging from 30 May 2016 to 1 January 2017) for the last event, suggesting the persistence of the initially introduced virus for the period of 2 years in which reported incidence was highest (2015 to 2016).

## DISCUSSION

To retrospectively explore the retrospective spatiotemporal evolution of ZIKV through the Midwestern Brazilian region, we generated 23 partial and near-complete genome sequences from the 2016 to 2018 ZIKV epidemic. Epidemiological data revealed that epidemic waves from the Brazilian Midwest region displayed their largest sizes between 2015 and 2017 (Fig. 1B). This was followed by a reduction in the number of reported cases over 2018 to 2021, likely a consequence of an expected, gradual accumulation of herd immunity, but the persistence of the initially introduced virus lineage through year-round transmission cycles was still indicated.

We found that the ZIKV epidemic in Goiás was ignited by multiple independent introduction events which we infer to have occurred between February 2014 and November 2016, most likely from northeastern and then later from southeastern Brazil, where the virus had already been circulating since late October 2013 (2, 5). Those findings are in line with previous studies that suggested that northeastern Brazil played a significant role in the establishment and dissemination of ZIKV in the Americas (2, 5, 18) and further reveal complex transmission dynamics within Brazilian regions. Since the first ZIKV confirmed case in Goiás was detected on 4 January 2015, our findings further highlight that the virus was cryptically circulated in this region for a period of 11 months, following a pattern that had been observed before during other Zika and dengue epidemics (5, 18).

In summary, our data reveal a complex pattern of ZIKV transmission between epidemic seasons, highlighting that the virus’s interregional spread might have been driven by a combination of several factors, including: (i) a completely susceptible population, (ii) favorable climatic conditions, and (iii) a sustained human mobility, as discussed elsewhere (7, 16). Together, those results highlight the utility of genomic, epidemiological, and evolutionary methods to understand mosquito-borne epidemics.

## MATERIALS AND METHODS

### Molecular screening.

Serum samples from 23 individuals presenting symptoms compatible with ZIKV infection were submitted to nanopore sequencing during a mobile genomic surveillance activity, which took place in Midwest Brazil in May 2019, under the scope of the ZIBRA-2 project (https://www.zibra2project.org/). Viral RNA was extracted and submitted to a real‐time PCR protocol adapted from reference 19 to confirm the previous diagnosis. Samples were selected for local sequencing based on a PCR cycle threshold (*C_T_*) of <32 to maximize genome coverage of clinical samples by nanopore sequencing (20) (Table S1).

### cDNA synthesis and multiplex tiling PCR.

Samples were submitted to a cDNA synthesis protocol described previously (20), a multiplex tiling PCR using Q5 high fidelity hot-start DNA polymerase (New England Biolabs), and a ZIKV sequencing primers scheme (20). The thermocycling conditions involved 40 cycles, and reaction conditions were as reported previously (20).

### Library preparation and nanopore sequencing.

Amplicons were purified using 1× AMPure XP beads, and cleaned-up PCR products concentrations were measured using Qubit dsDNA HS assay kit. DNA library preparation was carried out using the ligation sequencing kit and the native barcoding kit (NBD104 and NBD114, Oxford Nanopore Technologies, Oxford, UK) (20). Sequencing libraries were loaded into an R9.4 flow cell (Oxford Nanopore Technologies). In each sequencing run, we used negative controls to prevent and check for possible contamination with less than 2% mean coverage.

### Generation of consensus sequences.

Raw files were basecalled using Guppy, and barcode demultiplexing was performed using qcat. Consensus sequences were generated by *de novo* assembling using Genome Detective (https://www.genomedetective.com/) (21).

### Phylogenetic and Bayesian analysis.

The 23 new genomic sequences reported in this study were initially submitted to a genotyping analysis using the phylogenetic arbovirus subtyping tool, available at http://genomedetective.com/app/typingtool/zika (22). Genomic data generated in this study were aligned with a worldwide, larger, and updated data set of ZIKV genome sequences (*n* = 479). Sequences were aligned using MAFFT (23), and preliminary ML-tree was inferred using IQTREE2 (24). Prior to temporal analysis, our data set was also assessed for molecular clock signal in TempEst v1.5.3 (25) following the removal of any potential outliers that may violate the molecular clock assumption. To estimate a time-calibrated phylogeny, we used the Bayesian software package BEASTv.1.10.4 (26), with the Bayesian Skyline tree prior (27) with an uncorrelated relaxed clock and the lognormal distribution (28). Analyses were run in duplicate in BEASTv.1.10.4 (26) for 100 million Markov chain Monte Carlo (MCMC) steps, sampling parameters and trees every 10,000th step. Convergence of MCMC chains was checked using Tracer v.1.7.1 (29). Maximum clade credibility trees were summarized using TreeAnnotator after discarding 10% as burn-in.

### Epidemiological data assembly.

Data of weekly notified ZIKV cases in Brazil, available at the Sistema de Informação de Agravos de Notificação (SINAN) (https://portalsinan.saude.gov.br/), were supplied by Brazilian Ministry of Health and were plotted using the R software version 3.5.1.

### Data availability.

Newly generated ZIKV sequences have been deposited in GenBank under accession numbers OL423647 to OL423669.
